# Compressed *k*NN: K-Nearest Neighbors with Data Compression

**DOI:** 10.3390/e21030234

**Published:** 2019-02-28

**Authors:** Jaime Salvador–Meneses, Zoila Ruiz–Chavez, Jose Garcia–Rodriguez

**Affiliations:** 1 Facultad de Ingeniería, Ciencias Físicas y Matemática, Universidad Central del Ecuador, Quito 170129, Ecuador; 2 Computer Technology Department, University of Alicante, 03080 Alicante, Spain

**Keywords:** classification, KNN, compression, categorical data, feature pre-processing

## Abstract

The *k*NN (k-nearest neighbors) classification algorithm is one of the most widely used non-parametric classification methods, however it is limited due to memory consumption related to the size of the dataset, which makes them impractical to apply to large volumes of data. Variations of this method have been proposed, such as condensed KNN which divides the training dataset into clusters to be classified, other variations reduce the input dataset in order to apply the algorithm. This paper presents a variation of the *k*NN algorithm, of the type structure less NN, to work with categorical data. Categorical data, due to their nature, can be compressed in order to decrease the memory requirements at the time of executing the classification. The method proposes a previous phase of compression of the data to then apply the algorithm on the compressed data. This allows us to maintain the whole dataset in memory which leads to a considerable reduction of the amount of memory required. Experiments and tests carried out on known datasets show the reduction in the volume of information stored in memory and maintain the accuracy of the classification. They also show a slight decrease in processing time because the information is decompressed in real time (on-the-fly) while the algorithm is running.

## 1. Introduction

Discrete data compression is an interesting problem especially when compressed data is required to maintain the characteristics of the original data [1]. Most of the state-of-the-art classification methods require a large amount of memory and time making them unfeasible options for some practical applications in the real world [2].

In many datasets, the number of attributes (also called the dimension) is large, and many algorithms do not work well with datasets that have a high dimension because they require all information to be stored in memory prior to processing. Nowadays, it is a challenge to process datasets with a high dimensionality such as censuses carried out in different countries [3]. A census is a particularly relevant process and a vital source of information for a country [4]. The predominant characteristic of this type of information is that most of the data is of categorical type.

The problem of assigning a class to a dataset is a basic action in data analysis and pattern recognition, the task consists labeling an observation from a set of known variables [5].

Supervised learning is a part of machine learning (ML) which tries to model the behavior of some system. The supervised models are created from observations which consist of a set of input and output data. A supervised model describes the function which associates inputs with output [6].

In many cases, k-nearest neighbors (*k*NN) is a simple and effective classification method [7]. However, it presents two major problems when it comes to implementation: (1) it is a lazy learning method and (2) it depends on the selection of the value of *k* [8]. Other limitations present in this method corresponds to the high memory consumption which limits its application [9].

In this work a new method to classify information, using the *k*NN algorithm, on a compressed dataset is proposed. The method proposes to compress observations into packets of a certain number of bits, in each packet a certain number of attributes are stored (compressed) through operations at the bit level. This avoids having to reduce the size of the dataset [9,10] to avoid the memory problem.

An interesting feature of the proposed method is that the information can be decompressed, observation by observation in real time (on-the-fly), without the need to decompress all the dataset and carry out it into the memory.

As an application of the compression mechanism, this work proposed the implementation of the *k*NN algorithm that works with compressed data, we call this method “Compressed *k*NN algorithm”.

The rest of this document is organized as follows: Section 2 shows a brief introduction to data classification techniques focusing on the *k*NN algorithm, in Section 3 the datasets used in this work are described, in Section 4 a variation of the algorithm for working with compressed data is presented, in Section 5 some results obtained with the execution of the proposed algorithm are presented, and finally, Section 6 shows some conclusions and future work.

## 2. Background

This section describes, in general, the process of data classification focusing on the *k*NN method (the algorithm is also presented). In addition, some compression/encoding techniques are described, as well as the metrics used for categorical, numerical or mixed information.

### 2.1. KNN

The *k*NN algorithm belongs to the family of methods known as instance based methods. These methods are based on the principle that observations (instances) within a dataset are usually placed close to other observations that have similar attributes [11].

Given an observation from which you want to predict the class to which it belongs, this method selects the closest observations from the dataset in such a way to minimize the distance [12]. There are two types of *k*NN algorithms [10]:

- Structure less NN
- Structure based NN

Algorithm 1 defines the basic scheme of the *k*NN classification method (structure less NN) on a dataset with *m* observations.

There are variations of this algorithm in order to reduce the input dataset size [13]. For example we can cite stochastic neighbor compression (SNN) [14] that tries to compress the input dataset in order to obtain a sample of the data, or ProtoNN—compressed and accurate *k*NN for resource-scarce devices [15] that generates a subset of small number of prototypes to represent the input dataset.

**Algorithm 1:** The *k*-nearest neighbors (KNN) algorithm.

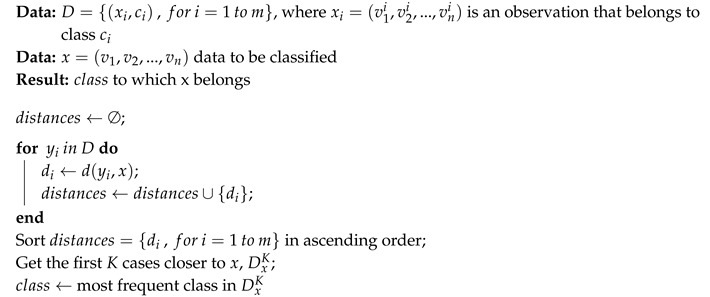



From the algorithm definition, it is necessary to define the concept of distance between observations.

#### 2.1.1. Categorical Data

In data mining there are several types of data, including numerical, categorical, text, images, audio, etc. Current methods that work with this type of information generally convert these types of data into numerical or categorical data [16].

Categorical or nominal data (also known as qualitative variables) have been the subject of studies in several contexts [17]. Data mining from categorical data is an important research topic in which several methods have been developed to work with this type of information such as decision trees, rough sets and others [16].

#### 2.1.2. Metrics

The similarity or distance between two objects (observations in our case) plays an important role in many tasks of classification or grouping of data [18].

In this study, the concept of similarity between two observations composed of categorical variables is considered.

The recommended distance function is the heterogeneous euclidean-overlap metric (HEOM) [19] which is defined as [20]:

(1) $$d\left(X,Y\right)=\sqrt{\sum_{i=1}^{n}d_{i}\left(x_{i},y_{i}\right)},$$

where $d_{i}\left(x_{i},x_{i}\right)$ is the distance between two observations in the *i*-th attribute.

In case that the *i*-th attribute has categorical values, it is recommended to use the Hamming distance [21], which is defined as:

(2) $$d_{0}\left(x_{i},y_{i}\right)=\left\{\begin{array}{ccc} 1 & if & x_{i}=y_{i}\\ 0 & if & x_{i}\neq y_{i} \end{array}.\right.$$

In case that the *i*-th attribute has numerical values, it is recommended to use the range normalized difference distance, which is defined as:

(3) $$d_{N}\left(x_{i},y_{i}\right)=\frac{|x_{i}-y_{i}|}{max\left(\left\{x_{i}\right\}\right)-min\left(\left\{x_{i}\right\}\right)}.$$

The above allows working with hybrid datasets, i.e., datasets composed of categorical and numerical variables.

In case the dataset only contains categorical variables, the Hamming distance is applied (see Equation (2)). If the dataset only contains numerical variables, it is possible to apply traditional distances such as Euclidean, Manhattan or Minkowski [21].

### 2.2. Data Compression

In database theory, a record *R* is a set of attributes $\left(f_{1},f_{2},\ldots ,f_{n}\right)$. An instance *r*, is a set of values associated with each attribute $f_{i}$, this is $\left(v_{1},v_{2},\ldots ,v_{n}\right)$ (values for each attribute). Each attribute $f_{i}$ has an associated $D_{i}$ set of possible values called the domain. Table 1 describes the above.

The main objective of this work is the classification of categorical data. Categorical data are stored in one-dimensional vectors (by rows or by columns) or in matrices depending on the information type.

From now on we will name $V_{i}$ to the set of values of the attribute $f_{i}$, that is:

$$V_{i}=\left[\begin{array}{c} v_{i}^{1}\\ v_{i}^{2}\\ v_{i}^{3}\\ .\\ .\\ .\\ v_{i}^{m-1}\\ v_{i}^{m} \end{array}\right]$$

We will name $R^{j}$ to the set of values of the *j*-th observation, this is:

$$R^{j}=\left(v_{1}^{j},v_{2}^{j},\ldots ,v_{n}^{j}\right).$$

Among the methods of compression/encoding of categorical data we can find [22]:

- Run-length encoding
- Offset-list encoding
- GNU ZIP (GZIP)
- Bit level compression

Each of these methods has advantages and disadvantages. In this work, the last option bit level compression is used, applied to the compression of observations $R^{j}$.

### 2.3. Bit Level Compression

In this section we review the mechanism for compressing categorical data, the compression method corresponds to a variation of the bit level compression used by the REDATAM software (http://www.redatam.org). This method doesn’t use all available bits because the block size may not be a multiple of the number of bits needed to represent the categories. Each data block stores one or more values depending of the maximum size in bits required to store the values [23].

Categorical data is represented as signed integer values of 32, 16 or 8 bits (4, 2, 1 bytes). This implies that to store a numerical value it is necessary to use 32 bits (or its equivalent in 2 or 1 byte). As an example, we will consider the case in which the information is represented as a set of four-bytes integer values (some frameworks use this representation [24]).

**Figure 1:**
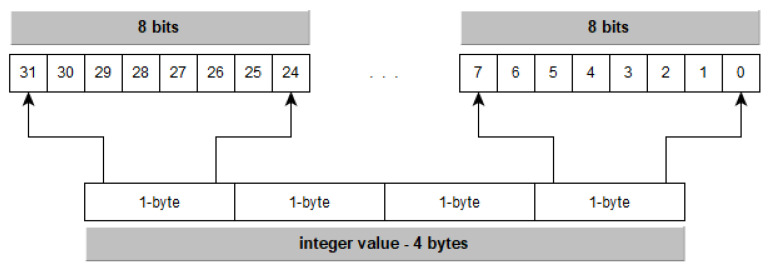
Representation of an integer value—4 bytes [22].

Figure 1 represents the bit distribution of a integer value composed of 4 bytes.

**Figure 2:**
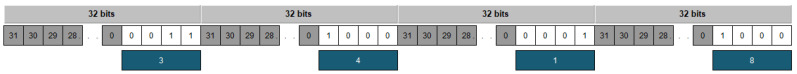
Example representation of four values [22].

Figure 2 shows a set of four values {3,4,1,8}, the gray area corresponds to space that is not used. Out of a total of 4×32=128 bits, only 16 are used, which represents 12.5% of the total storage used. We can make a similar representation using different block sizes.

**Figure 3:**
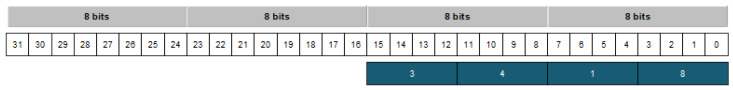
Compressed representation of four values.

The bit level compression can be implemented using 64, 32, 16 or 8 bits per block, these sizes correspond to the computer internal representation of integer values. Figure 2 shows the storage using 32 bits per block, the gray areas (unused bits of Figure 2) are used to store additional values. In one 32-bit block, we can store the four values {3,4,1,8} reducing the memory consumption to 12.4% of the original size. Figure 3 shows one 32-bit block that store all the four values.

Table 2 shows the number of bits used to represent a group of categorical values. First column shows the number of categories to represent (two, between three and four, between five and eight, etc.), the second column shows the number of bits needed to represent the categories mentioned, the third and fifth columns show the total number of elements that can be stored within 32 or 64 bits per block and, finally, the fourth and sixth columns show the number of bits that we lose using 32 or 64 bits per block.

The compression implemented use a fixed number of bits for all the data in order to reduce the number of operations, this may cause that a group ob bits may be lost (in the case that the number of bits is not a divisor of 64, 32, 16 or eight).

For example, if we need to compress eight categories using a 64-bit block, we lose 64−3×21=1 bit in each block. So, in order to get a better compression ratio and avoid as much as possible the lost bits, we should choose the biggest block size.

## 3. Datasets

This section describes the datasets used in this work as well as some of their characteristics.

Datasets are generally stored as flat files. Rows represent objects (also called records, observations, points) and columns represent attributes (features) [25]. Two test datasets were used in the experiments:

- Census income data set (CIDS), which represents a mixed dataset containing both categorical and numeric variables.
- Wisconsin breast cancer (original) (WBC-original) which represents a dataset containing only categorical variables.

Table 3 shows a brief description of the two datasets.

Dataset selection was made to show how the algorithm works with a dataset containing only categorical variables and with a mixed dataset (categorical and numeric variables).

Each of the datasets used is described below.

### 3.1. Census Income Data Set

The Census Income Data Set (https://archive.ics.uci.edu/ml/datasets/census+income) is used to test classification algorithms. The purpose is to predict whether a person earns more than $50,000 a year.

Table 4 describes the type and range of each variable.

The dataset contains 32,561 observations of which 2399 observations contain missing values (NA). In order to simplify the process, all observations containing at least one NA value were deleted, so that the resulting dataset contains 30,162 observations.

As can be seen in the table above, there are variables that are originally considered continuous. However, they can be treated as categorical because they take integer values within a small range. These variables are considered categorical in the tests performed.

### 3.2. Wisconsin Breast Cancer (Original)

The Wisconsin breast cancer (original) (https://archive.ics.uci.edu/ml/datasets/breast+cancer+wisconsin+(original)) dataset is used to test classification algorithms. The purpose is to predict malignant (carcinogenic) observations of benign (non-cancerous) observations [26].

Table 5 describes the type and range of each variable.

The dataset contains 699 observations of which 16 observations contain missing values (NA). In order to simplify the process, all observations containing at least one NA value were deleted, so that the resulting dataset contains 683 observations.

As seen in Table 5, the sample code number variable corresponds to the identifier of the observation so it can be discarded. The rest of the attributes are all categorical in the range of one to 10, with the exception of the class variable that has two possible values; 2 or 4.

## 4. Compressed *k*NN

This section proposes a variation of the *k*NN algorithm to work with compressed categorical data. The proposed compression method uses a variation of the method described in [22] for which it is proposed to compress each observation $R^{j}$ with a bit schema. Similar to the scheme used by the REDATAM 7 V3 software [23], each block stores the values corresponding to $v_{1}^{j},v_{2}^{j},\ldots ,v_{n}^{j}$.

To apply the proposed scheme, it is necessary to determine the maximum number of bits to represent all possible bits of each observation. This imposes that, prior to the process, the maximum number of bits needed to represent each attribute ($f_{i}$) is determined to finally define the maximum of these values.

If we define $N_{i}$ as the number of bits necessary to represent the attribute (variable) $f_{i}$, the number of bits necessary to represent an observation would be given by:

(4) $$N=n*M,\;where\;M=max\;\{N_{i};i=1,2,\ldots ,n\}.$$

Also note that we use the same $N_{i}$ value, defined as $N_{i}=M$, for all variables in order to avoid additional operations in the decompression/decoding phase.

The proposed method uses bit-wise operations (AND, OR, etc.) which are an important part of modern programming languages. These operations are widely used because they allow arithmetic operations to be replaced by more efficient operations [27].

**Figure 4:**
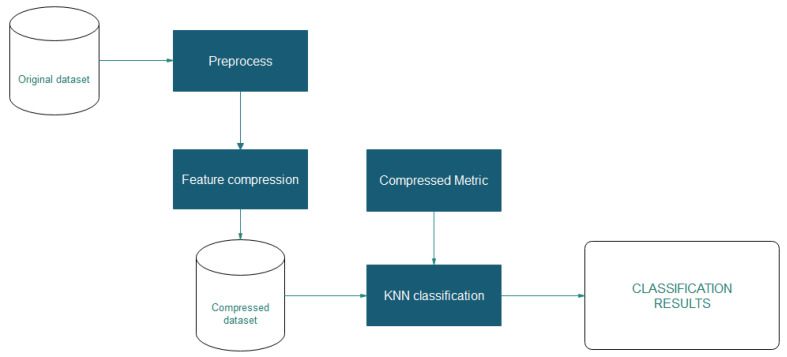
Proposed compression and classification schema.

Figure 4 describes the phases of the proposed method that can be summarized as:

1. Preprocess: In this phase, starting from the original dataset, the possible categorical and numerical variables are determined, as well as the ranges of each categorical variable.
2. Feature compression: In this phase, the compression algorithm is applied to the original dataset.
3. *k*NN Classification: In this phase the classification is applied on the compressed dataset.

### 4.1. Preprocess

This phase determines the number of bits needed to represent each categorical variable and then determine the value of *N* given by Equation (4). Let’s take the WBC dataset as an example, in a first step the sample code number (v1, see Table 5) variable was eliminated.

Records containing missing values were then deleted, resulting in a dataset of 683 observations. Two datasets were generated, one for training (80% of the data) and one for testing (20% of the data). These two datasets constitute the input data for compression and then for classification (see Table 6).

The minimum amount of bits used to represent a range of categories ($N_{i}$) and the elements per block ($N_{V}$) can be obtained using:

(5) $$N_{i}=\lceil \frac{ln(x_{k})}{ln(2)}\rceil ,\;\;\;N_{V}=\lfloor \frac{BlockSize}{M}\rfloor ,$$

where BlockSize represents the size of the block and can be eight, 16, 32 or 64, ⌈·⌉ represents the smallest integer greater than or equal to its argument and is defined by ⌈x⌉=min{p∈Z|p⩾x} and ⌊·⌋ represents the biggest integer smaller than or equal to its argument and is defined by ⌊x⌋=max{p∈Z|p⩽x} [22].

In the rest of this document, as an example, we use a block size equal to 32, that is BlockSize=32.

All variables have a range from one to 10 (10 categories), so the number of bits needed for compression corresponds to $N_{i}=4$ (4 bits) and the number of elements per block corresponds to $N_{V}=32/4=8$ (with a 32-bit block), which in this case indicates that eight values can be stored in a block, which corresponds to the values of variables two through nine, the remaining variable (variable number 10) must be stored in an additional block.

A similar process was made with the census income data set. All categorical variables have a range from one to 128, so the number of bits needed for compression correspond to N=7 (7 bits). After the elimination of the NA values, the final dataset has 30,162 observations. The final dataset was split in two; 80% for training and 20% for testing.

### 4.2. Feature Compression

**Figure 5:**
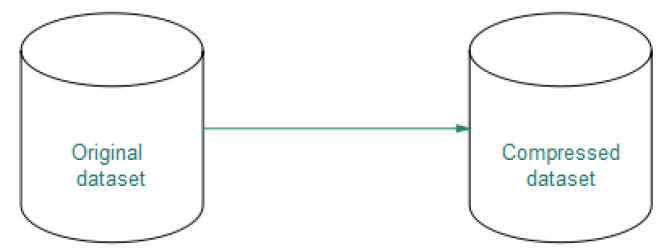
Proposed compression and classification schema.

In this phase we proceed to compress each observation using a total number of bits equal to *M* (see Equation (4)), with this, in 32 bits block it is possible to store the values of 32/M variables (attributes). The process start with the original dataset to generate a compressed/encoded dataset (see Figure 5).

If *n* is the total number of attributes (see Table 1), the number of blocks needed to represent all the attributes corresponds to [22]:

(6) total=⌈n∗M/BlockSize⌉.

If we use a BlockSize equals to 32, Equation (7) can be written as:

(7) total=⌈n∗M/32⌉.

In the case of the WBC dataset, total=⌈9∗4/32⌉=2. This means that the original dataset can be represented by two one-dimensional vectors, the first containing the information of eight variables and the second containing the information of the remaining variable.

The blocks w1 and w2 are defined in such a way that w1 contains the compressed/encoded information of the variables $\left\{v_{2},v_{3},v_{4},v_{5},v_{6},v_{7},v_{8},v_{9}\right\}$ and w2 contains the compressed/encoded information of the variables $\left\{v_{10}\right\}$. The new generated dataset is called *WBC encoded dataset* (see Table 7).

In the case of the CID dataset, total=⌈(14−3)∗7/32⌉=3. This means that the original dataset can be represented by six one-dimensional vectors, the first three contains the information of the variables $\left\{v_{1},v_{2},v_{4},v_{5},v_{6},v_{7},v_{8},v_{9},v_{10},v_{13},v_{14}\right\}$ and the rest of the vectors (the last three) contains the information of the remaining uncompressed variables $\left\{v_{3},v_{11},v_{12}\right\}$.

The blocks w1, w2 and w3 are defined in such a way that w1 contains the compressed/encoded information of the variables $\left\{v_{1},v_{2},v_{4},v_{5}\right\}$, w2 contains the compressed/encoded information of the variables $\left\{v_{6},v_{7},v_{8},v_{9}\right\}$ and w3 contains the compressed/encoded information of the variables $\left\{v_{10},v_{13},v_{14}\right\}$. The new generated dataset is called CID encoded dataset (see Table 8).

**Figure 6:**
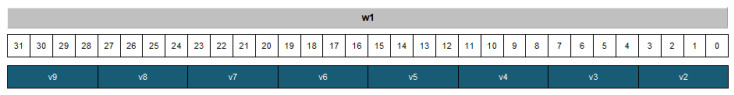
Compression schema.

In [22] the compression algorithm was applied to the columns of the dataset, in this work we apply the algorithm to the rows (observations) of the dataset. Figure 6 describes the compression of w1 in the WBC dataset.

### 4.3. *k*NN Classification

**Figure 7:**
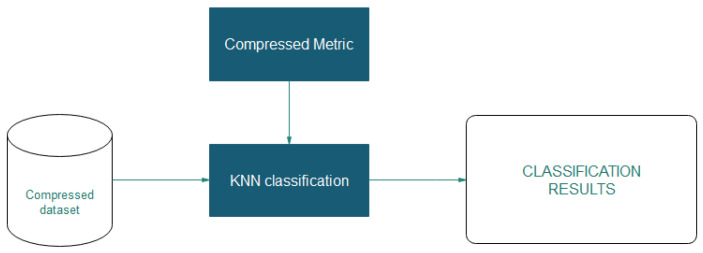
The *k*-nearest neighbours (*k*NN) classification.

The classification process uses the compressed dataset obtained by applying the compression of the data over the original dataset as the input dataset. Figure 7 shows the implemented classification process.

The process uses custom metrics to work with the compressed data, and in this case the HEOM and Hamming metric are used. At the classification stage, it is necessary to decompress the information and calculate the distances between observations.

The distance calculation uses the algorithm described in [28] for the calculation of the $L^{2}$ norm modified to decompress two vectors at a time (the observations from which the distance is calculated).

Because the execution of the *k*NN algorithm compares an observation with all other observations, a local cache is used to avoid repeated decompression of the first vector (vec1). Algorithms 2 and 3 describes the calculation of Euclidean and Hamming distances with on-the-fly decompression.

**Algorithm 2:** Euclidean compressed_distance: distance between two compressed vectors.

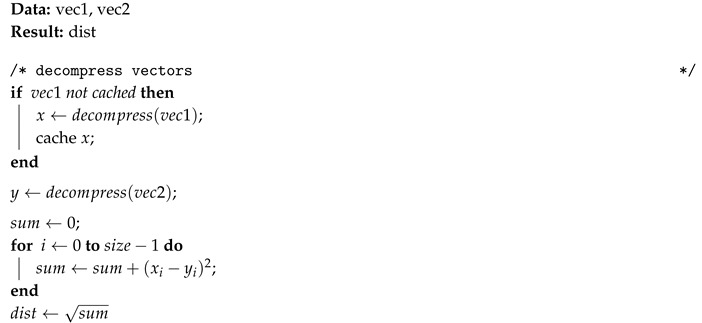



**Algorithm 3:** Hamming compressed_distance: distance between two compressed vectors.

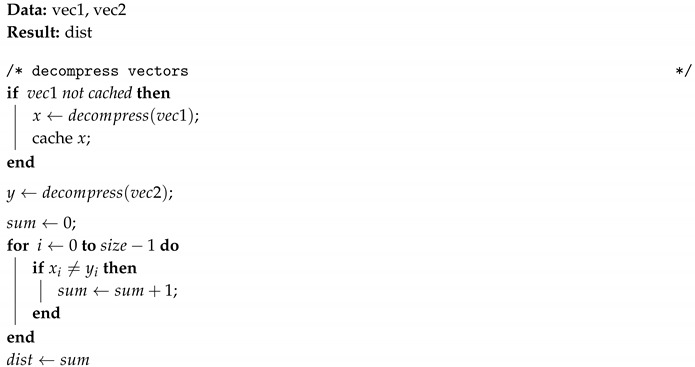



Algorithm 4 describes the calculation of Hamming distances without decompression using bit-wise operations.

**Algorithm 4:** Hamming compressed_distance without decompression: distance between two compressed vectors.

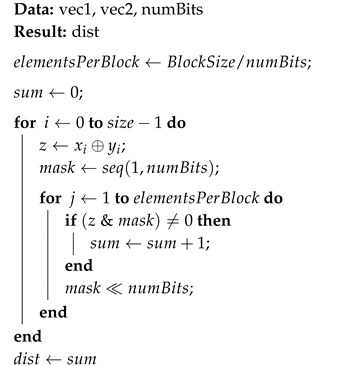



Similarly, it is possible to define algorithms to calculate other metrics.

After defining the metric we proceed to define the *k*NN algorithm on compressed data. Algorithm 5 describes the Compressed-*k*NN algorithm. 

**Algorithm 5:** Compressed *k*NN.

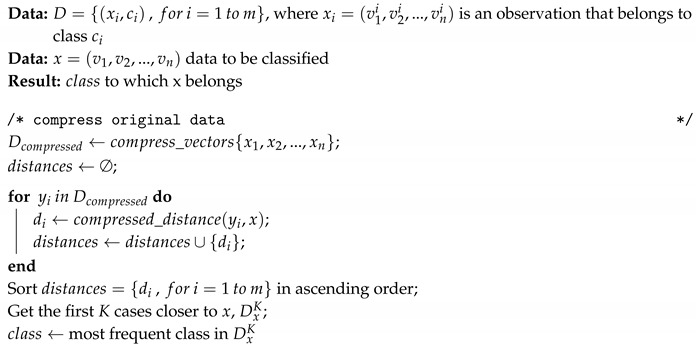



The first step is to compress the original dataset, then iterate over the compressed dataset and calculate the distances between the observation to be classified and the dataset elements. Finally, the set of distances $d_{i}$ is ordered in ascending order and the most repeated class of the *K*-first elements is selected.

The algorithm can be generalized to work with other *k*NN implementations, the paper presents results obtained from two variations of the algorithm:

- Linear search: brute force linear nearest neighbor search.
- Cover search: a tree data structure for fast nearest neighbor operations in general n- point metric spaces [29].

In both cases, we use the distance described in Section 2.1.2.

As we can see, the principal advantage of the proposed algorithm is the reduction of the dataset size. The result of the compression phase is a dataset that can be fully loaded into memory thus reducing the memory limitations described in Section 1 without sampling or dimension reduction. If we combine compression with sampling or dimension reduction, we can obtain a better algorithm than the described in Algorithm 5 (this is proposed as a future work in Section 6).

#### Limitations

There are some limitations related to distance calculation. Prior to calculating the distance, the entire observation must be decompressed. If we use a dataset only with categorical variables, we can use Algorithm 4 in order to calculate the Hamming distance without decompression.

If we try to classify a group of data (test dataset), for each test observation, we need to decompress the train observations, so if we have a *t* observations in test dataset, we need to decompress *t*—times each train observation. If the test dataset is also compressed, to avoid the limitation showed above, Algorithms 2 and 3 use a local cache to store the uncompressed test observation. We can extend the cache idea to the training dataset and use a small local-cache of uncompressed training data.

Another limitation is related with the range that categorical variables may have. If the range is big, we can only compress a few values inside of a block, so the calculations needed to decompress may lift up the processing time.

## 5. Experimental Results

This section presents some results obtained by applying the *k*NN algorithm on the original and on the compressed dataset. Similarly, the tests were performed using two metrics: Hamming and HEOM (for uncompressed and compressed values).

All tests were performed using the statistical machine intelligence and learning engine (SMILE) framework (https://haifengl.github.io/smile/) modified to work with compressed data according to the algorithm described in Section 2.3. See the source code in the Supplementary. The algorithm used corresponds to *k*NN with a value of *k* between five to 20 (k=5,10,15,20), which by default uses an Euclidean metric EuclideanDistance that operates with real (double) numbers.

The WBC dataset was compressed using a number of bits equal to four (see Equation (4)). The CID dataset was compressed with a mixed schema using a number of bits equal to seven (see Equation (4)).

Table 9 shows the execution times of the classification and cross validation (*k*-fold=10) for different values of *k* with linear search. In the case of WBC dataset, the Hamming metric is used.

As can be seen, the accuracy of the classification is the same in both cases (uncompressed dataset and compressed dataset) as expected. The classification time is slightly shorter when working with the compressed dataset (see Table 9).

Table 10 shows the execution times of the classification and cross validation (*k*-fold=10) for different values of *k* with linear search. In the case of CID dataset, the HEOM metric is used.

As can be seen, the accuracy of the classification is the same in both cases (uncompressed dataset and compressed dataset). The classification time is slightly shorter when working with the compressed dataset (see Table 10).

### 5.1. Test Platform

The platform on which the tests were carried out corresponds to:

- Processor: Intel(R) Core(TM) i5-5200 CPU
- Processor speed: 2.20 GHz
- RAM Memory: 16.0 GB
- Operating System: Windows 10 Pro 64 bits
- Compiler: JDK 1.8.0.144 64-Bit Server VM
- Machine Learning Framework: SMILE 1.5.2

### 5.2. Memory Consumption

The memory consumption represents the amount of memory required to represent the complete dataset. Table 11 shows a summary of the datasets used in the experiments carried on.

Table 12 shows the amount of memory used to represent both datasets (WBC and CID) using a traditional vector representation using *int*, short and byte as element type. Column % Memory is calculated using the int representation as 100%. Due to final weight variable has values between 12,285 and 1,484,705 (see Table 4), CID datasets only are represented as a vector of integer values.

Table 13 shows the amount of memory used to represent WBC dataset using different block sizes. Column % Memory is calculated using the int vector size as 100% (see Table 12).

Table 14 shows the amount of memory used to represent CID dataset using different block sizes. Column % Memory is calculated using the int vector size as 100% (see Table 12). Due to *final weight* variable has values between 12,285 and 1,484,705 (see Table 4), CID datasets only are compressed with 32 and 64 bits.

The *WBC* dataset contains nine valid variables and 683 observations. Since four bits are used for compression, this means that a 32-bit block compresses four attributes, so two 32-bit blocks are needed to represent the 9 categorical attributes.

With this data, the memory size to represent the complete dataset corresponds to the product of the total number of elements of the dataset by the number of attributes by the size of the representation of an integer value (sizeof(int)):

(8) Numbytes=683∗9∗4=24,588bytes(forWBCdataset)

While the value of the representation in the compressed format corresponds to the product of the total number of elements of the compressed dataset by the number of attributes (blocks, see Equation (6)) resulting from the compression by the size of the representation of an integer value (sizeof(int)):

(9) Numbytes=683∗2∗4=5464bytes(forWBCdataset)

**Figure 8:**
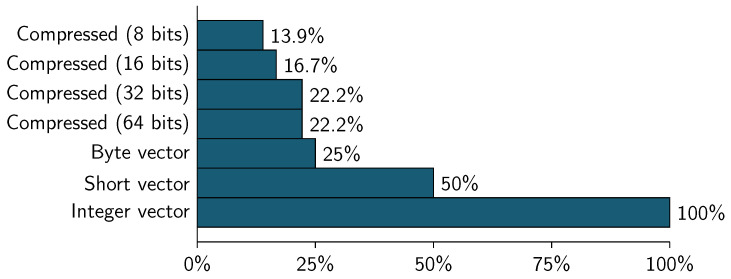
Wisconsin breast cancer (WBC)—dataset size by memory consumption.

**Figure 9:**
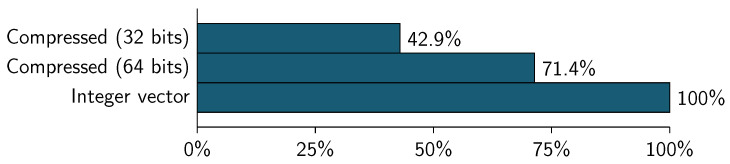
Census income dataset (CID)—dataset size by memory consumption.

Taking as 100% the value of the uncompressed representation (Equation (8)), Figure 8 and Figure 9 show the memory consumption for the datasets using different vector representations and block sizes (for compression).

As can be seen, the memory consumption for WBC dataset is slower using any compressed representation (8, 16, 32 or 64 bits). The best compression ratio can be obtained using a 8-bit block size, this represent the 13.9% of the integer vector representation and a 55.6% of the byte vector representation.

In the case of CID dataset, the compressed representation is slower than the traditional representation. Using 32-bit block, the compressed format represents the 42.9% of the vector representation.

**Figure 10:**
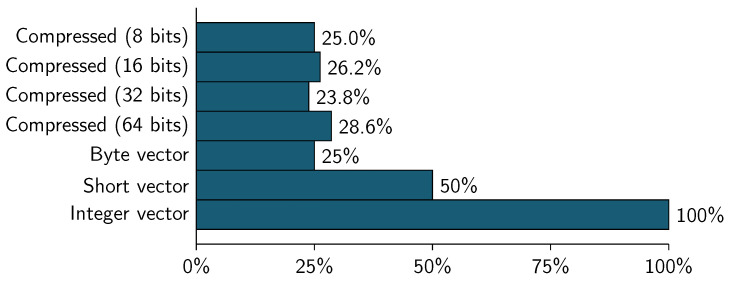
Base de Datos-Censo de Población y Vivienda 2010—Dataset size by memory consumption.

Table 15 and Table 16, and Figure 10 show the compression of a real world census data taken from Base de Datos-Censo de Población y Vivienda 2010 (http://www.ecuadorencifras.gob.ec/base-de-datos-censo-de-poblacion-y-vivienda-2010/). The dataset is a subset of the original census used for imputation of missing data [30].

The compressed representation uses 6 bits to represent the data, so we can compress/encoded 5 values in each 32-bit block, this lead to have a new dataset (compressed) with 5 columns instead of 21 (the original).

As can be seen, the total memory used to represent the original dataset is 1.2166 GBytes (using a vector that contains integer values) and the compressed representation is equivalent to 32.81% of the original dataset. The compression using 32-bit block is slower than any vector representation.

### 5.3. Accuracy

**Figure 11:**
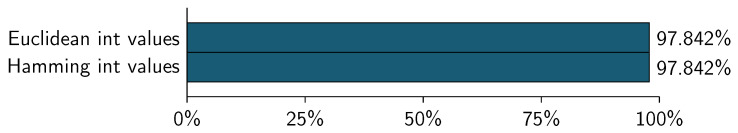
WBC—precision by metric.

**Figure 12:**
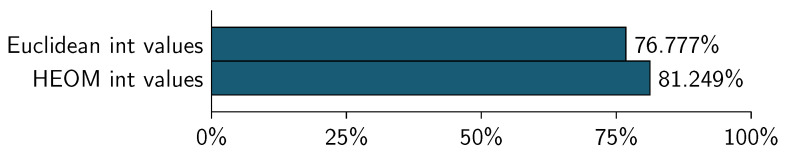
Census income dataset—precision by metric.

Figure 11 and Figure 12 show the accuracy comparison for the classification using the following metrics:

- Euclidean metric, observations with integer values.
- HEOM metric, observations with integer values (see Section 2.1.2).

In all cases the version of the compressed datasets was used with a k=5. In the case of the WBC dataset, the HEOM metric corresponds to the Hamming metric since all variables are categorical.

As can be seen in the figure, the HEOM metric provides a better result. The result for execution with compression is the same for execution without compression since the same metric is used, what varies is the processing speed.

### 5.4. Processing Speed

**Figure 13:**
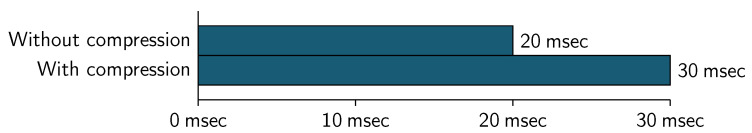
WBC—Time for cross-validation *k*-fold=10.

**Figure 14:**
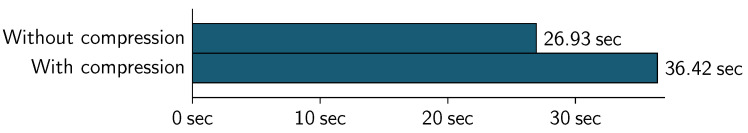
CID—Time for cross-validation *k*-fold=10.

Figure 13 and Figure 14 show the comparison in the cross-validation speed. The *k*NN algorithm was executed with both uncompressed and compressed datasets (using a 32-bit block) a value of *k*-fold=10 and k=5.

As can be seen in the figures, the use of compression show an increase in the processing time.

### 5.5. *k*NN Variations

This section presents some results obtained by applying the *k*NN algorithm on the compressed dataset with a Cover Tree search algorithm.

Table 17 shows the execution times of the classification for different values of *k* using a linear search and a cover tree search. In the case of WBC dataset, the Hamming metric was used.

Table 18 shows the execution times of the classification for different values of *k* using a linear search and a cover tree search. In the case of CID dataset, the HEOM metric was used.

## 6. Conclusions

In this paper we reviewed one of the most widely used classification algorithms, the *k*NN. Along with some traditional methods of compression/encoding of categorical data a model called “compressed *k*NN” was proposed to work directly on compressed categorical data. After performing some tests on known datasets (WBC and CID) we can conclude that the proposed method considerably reduces the amount of memory used by the *k*NN algorithm and in turn maintains the same percentage of error classification made with the original method.

The inclusion of the compression stage prior to the execution of the algorithm guarantees a decrease in the percentage of memory needed to represent the whole dataset (see Figure 8 and Figure 9). The inclusion of the distance calculation for compressed categorical data (see Algorithm 2) slightly increases the classification time because it is necessary to decompress the observations prior to the distance calculation to determine the nearest neighbors. This paper also provides an algorithm for Hamming Distance without decompression.

Future works include (1) the implementation of variations of the *k*NN algorithm of the type structure less NN and structure based NN, (2) extending the concepts of compression to cluster algorithms, (3) the implementation of hybrid algorithms that uses sampling with compression, and (4) reordering attributes before compression.

## Figures and Tables

**Table 1:** Dataset representation.

Observation	$f_{1}$	$f_{2}$	$f_{3}$	...	$f_{n}$
1	$v_{1}^{1}$	$v_{2}^{1}$	$v_{3}^{1}$	...	$v_{n}^{1}$
2	$v_{1}^{2}$	$v_{2}^{2}$	$v_{3}^{2}$	...	$v_{n}^{2}$
3	$v_{1}^{3}$	$v_{2}^{3}$	$v_{3}^{3}$	...	$v_{n}^{3}$
...	...	...	...	...	...
m−1	$v_{1}^{m-1}$	$v_{2}^{m-1}$	$v_{3}^{m-1}$	...	$v_{n}^{m-1}$
*m*	$v_{1}^{m}$	$v_{2}^{m}$	$v_{3}^{m}$	...	$v_{n}^{m}$

**Table 2:** Amount of elements to represent.

Total Categories	Total Bits	Total Elements (32-Bits)	Lost Bits	Total Elements (64 Bits)	Lost Bits
2	1	32	0	64	0
3–4	2	16	0	32	0
5–8	3	10	2	21	1
9–16	4	8	0	16	0
17–32	5	6	2	12	4
33–64	6	5	2	10	4
65–128	7	4	4	9	1
129–256	8	4	0	8	0
257–512	9	3	5	7	1

**Table 3:** Datasets description.

Dataset	No. Attributes	No. Observations	No. Classes
Census Income Data Set	15	32,561	2
Wisconsin Breast Cancer (original)	11	699	2

**Table 4:** Census income data set.

No.	Attribute	Original Type	Range	Type Used
1	age	continuous	17–90	categorical
2	workclassge	categorical	1–8	categorical
3	final weight (fnlwgt)	continuous	12,285–1,484,705	numeric
4	education	categorical	1–16	categorical
5	education-num	continuous	1–16	categorical
6	marital-status	categorical	1–7	categorical
7	occupation	categorical	1–14	categorical
8	relationship	categorical	1–6	categorical
9	race	categorical	1–5	categorical
10	sex	categorical	1–2	categorical
11	capital-gain	continuous	0–99,999	numeric
12	capital-loss	continuous	0–4356	numeric
13	hours-per-week	continuous	1–99	categorical
14	native-country	continuous	1–41	categorical
15	class	categorical	1–2	categorical

**Table 5:** Wisconsin breast cancer (original).

No.	Attribute	Type	Range
1	Sample code number	id	
2	Clump Thickness	categorical	1–10
3	Uniformity of Cell Size	categorical	1–10
4	Uniformity of Cell Shape	categorical	1–10
5	Marginal Adhesion	categorical	1–10
6	Single Epithelial Cell Size	categorical	1–10
7	Bare Nuclei	categorical	1–10
8	Bland Chromatin	categorical	1–10
9	Normal Nucleoli	categorical	1–10
10	Mitoses	categorical	1–10
11	Class	categorical	2 = benign, 4 = malignant

**Table 6:** Wisconsin breast cancer (WBC) input dataset.

$v_{2}$	$v_{3}$	$v_{4}$	$v_{5}$	$v_{6}$	$v_{7}$	$v_{8}$	$v_{9}$	$v_{10}$
.	.	.	.	.	.	.	.	.
.	.	.	.	.	.	.	.	.
.	.	.	.	.	.	.	.	.
.	.	.	.	.	.	.	.	.

**Table 7:** WBC encoded dataset.

w1	w2
.	.
.	.
.	.
.	.

**Table 8:** Census income dataset (CID) encoded dataset.

w1	w2	w3	$v_{3}$	$v_{11}$	$v_{12}$
.	.	.	.	.	.
.	.	.	.	.	.
.	.	.	.	.	.
.	.	.	.	.	.

**Table 9:** WBC—processing speed (linear search).

	Compressed Dataset		Uncompressed Dataset	
*k*	Time (ms)	Accuracy (%)	Time (ms)	Accuracy (%)
5	30	95.404	18	95.386
10	40	94.669	19	94.301
15	42	94.118	19	94.136
20	50	93.934	18	93.548

**Table 10:** CID—processing speed (linear search).

	Compressed Dataset		Uncompressed Dataset	
*k*	Time (ms)	Accuracy (%)	Time (ms)	Accuracy (%)
5	36.42	81.712	26.93	81.712
10	40.92	82.528	29.37	82.528
15	39.12	82.902	27.49	82.902
20	40.26	82.955	27.16	82.955

**Table 11:** Dataset summary

Datset	Obervations	Attributes	Bits for Compression
WBC	683	9	4
CID	30,162	14	7

**Table 12:** Dataset representation with different vector types.

Vector Type	WBC		CID	
	Memory (bytes)	% Memory	Memory (bytes)	% Memory
int	24,588	100.0	1,689,072	100.0
short	12,294	50.0	-	-
byte	6147	25.0	-	-

**Table 13:** WBC dataset compression with different block size.

Block Size (bits)	Total Blocks	Memory (bytes)	% Memory
64	1	5464	22.2
32	2	5464	22.2
16	3	4098	16.7
8	5	3415	13.9

**Table 14:** CID dataset compression with different block size.

Block Size (bits)	Total Blocks	Memory (bytes)	% Memory
64	5	1,206,480	71.4
32	6	723,888	42.9

**Table 15:** Base de Datos-Censo de Población y Vivienda 2010.

Observations	Attributes	Bits for Compression	Memory (GBytes) (Integer Vector)
14,483,499	21	6	1.2166

**Table 16:** Censo de Población y Vivienda 2010—compression with different block size.

Block Size (bits)	Total Blocks	Memory (GBytes)	% Memory
64	3	0.3476	28.6
32	5	0.2897	23.8
16	11	0.3186	26.2
8	21	0.3042	25.0

**Table 17:** WBC—Processing speed.

	Classification Linear Search		Classification Cover Tree Search	
*k*	Time (ms)	Accuracy (%)	Time (ms)	Accuracy (%)
5	30	95.404	120	95.315
10	40	94.669	150	94.143
15	42	94.118	175	93.997
20	50	93.937	180	93.119

**Table 18:** CID—Processing speed.

	Classification Linear Search		Classification Cover Tree Search	
*k*	Time (ms)	Accuracy (%)	Time (ms)	Accuracy (%)
5	36.42	81.712	40.81	81.500
10	40.92	82.528	44.20	82.541
15	39.12	82.902	50.36	82.717
20	40.26	82.955	51.97	82.823

## Supplementary Materials

The source code is available online at https://github.com/jsalvador2k14/knn.

## Abbreviations

The following abbreviations are used in this manuscript:

CID	Census income dataset
CIDS	Census income data set
GZIP	GNU ZIP
KNN, *k*NN	K-nearest neighbors
HEOM	Heterogeneous euclidean-overlap metric
ML	Machine learning
NA	Not applicable
NN	Nearest neighbors
RAM	Random access memory
SMILE	Statistical machine intelligence and learning engine
WBC-original	Wisconsin breast cancer (original)
